# Occurrence of Antimicrobial Resistance in *Kocuria rhizophila* Bacteria Isolated From *Salmo munzuricus* (Teleostei: Salmonidae) Samples in Natural Resources

**DOI:** 10.1002/mbo3.70354

**Published:** 2026-06-29

**Authors:** Bülent KAR

**Affiliations:** ^1^ Munzur University, Tunceli Vocational School, Department of Organic Agriculture Tunceli Center/Tunceli Turkey

**Keywords:** antimicrobial resistance, aquatic ecosystem, *Kocuria rhizophila*, molecular identification, *Salmo munzuricus*

## Abstract

Global warming and environmental pollution trigger the emergence of antimicrobial‐resistant (AMR) opportunistic pathogens in the aquatic microflora. This study aimed to determine the characterization and AMR profile of the opportunistic pathogen *Kocuria rhizophila* isolated from the tissues of *Salmo munzuricus* (Munzur trout), an endemic species. Samples collected from the Munzur River were identified using morphological and molecular (16S rDNA) methods; bacterial isolates were characterized by MALDI‐TOF and sequencing. The resistance status against 11 different antibiotics was analyzed with the Kirby‐Bauer method. The findings revealed that the isolates (with 99.51% accuracy as *K. rhizophila*) showed full resistance to Penicillin G (*p* < 0.01) and were moderately sensitive to gentamicin and tetracycline. High sensitivity to amoxicillin and ampicillin was detected. These findings demonstrate that even in natural environments, the fish microbiome in wild endemic populations can be susceptible to the development of AMR in response to environmental changes. Therefore, determining the resistance of opportunistic pathogens in wild fish species such as *Salmo munzuricus* to different classes of antibiotics and studying their spread is extremely important for natural ecosystems.

## Graphical abstract

This study examined the antibiotic susceptibility of Kocuria rhizophila isolated from *Salmo munzuricus* samples taken from the Munzur River. The isolates are resistant to Penicillin G, moderately susceptible to gentamicin and tetracycline, and highly susceptible to amoxicillin and ampicillin. The findings indicate resistance development depending on environmental factors.

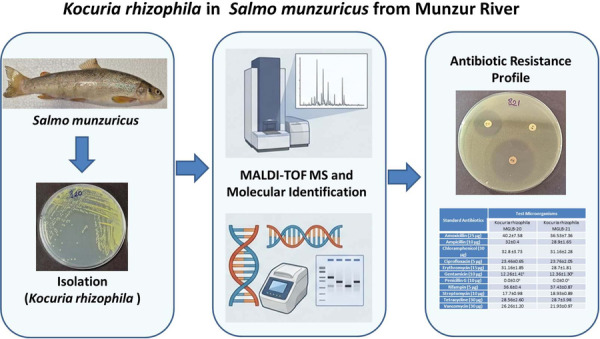

## 1 Introduction

Global warming and environmental pollution have begun to affect the microflora of fish, as they do in many other living organisms (Okon et al. 2024). This interaction not only alters the existing microbial flora but also changes the general characteristics of the microorganisms (Naga et al. 2025). For example, changes in the microbial flora of the host, or an increase in the number of microorganisms in the existing flora, manifest as molecular and genetic changes in the microorganisms (Chen et al. 2024). These changes generally benefit the microorganisms while harming the host. One of the most striking aspects of this is microbial resistance in microorganisms. Just as in fish, antimicrobial resistance (AMR) developing in many organisms in their natural environment is closely linked to human activities and the spread of resistant bacteria into the environment (Larsson and Flach 2022). This pollution, originating from aquaculture systems or other environmental sources, leads to the emergence and spread of antimicrobial resistance in fish in natural water systems and in bacteria within the aquatic ecosystem (Cherian et al. 2023; Milijasevic et al. 2024). Common fish pathogens reported in studies to date include those from the genera Vibrio, Aeromonas, and Edwardsiella (Maiti et al. 2020; Deekshit et al. 2023). These species can also cause morbidity and mortality in natural fish populations (Deekshit et al. 2023). In addition, many different bacterial taxa occur naturally in aquatic environments. Global warming and sudden environmental changes are known to cause diseases in fish that are stressed or weakened by unexpected effects (Deekshit et al. 2023). Therefore, the emergence of numerous opportunistic pathogens that can cause disease is inevitable.

The name *Kocuria* comes from the Slovak microbiologist Miroslav Kocúr (Sarojini et al. 2025). The genus *Kocuria*, the bacteria of the genus *Kocuria* belonging to the family Micrococcaceae; finds Gram positive, catalase positive, coagulase negative, coccus shaped, generally as commensal in soil, water, and human skin (Azevedo et al. 2025). These bacteria, which were initially classified as *Micrococcus*, were reclassified based on 16S rDNA sequencing results and the amino acid composition in the structure of peptidoglycan (Stackebrandt et al. 1996). These organisms can be seen in irregular clusters, pairs, short chains, or tetrads. Today, 26 *Kocuria* species have been identified (Sarojini et al. 2025). K. rhizophila is also one of these species. *K. rhizophila*; is a *Kocuria* bacterial species that does not form endospores, is seen in clusters with a diameter ranging between 0.5 and 0.75 μm, and is not acid resistant (Sarojini et al. 2025). Although *Kocuria rhizophila* is known to constitute the physiological microflora of trout intestines, recent studies have reported that it is an opportunistic bacterial species that can cause abnormal deaths and pathological changes in internal organs and external tissues (Kim et al. 2007; Pękala et al. 2018; Zaheen et al. 2022). *Kocuria rhizophila*'s ability to metabolize plant material is quite important; this is a significant advantage for vital activities in environments rich in decaying organic matter, such as aquatic systems (Takarada et al. 2008). The ability of this species to develop in challenging ecological niches increases its acquisition of resistance against selective pressures such as environmental pollutants and antimicrobials, and makes its evaluation in terms of antimicrobial resistance mandatory (Azevedo et al. 2025). The ability to thrive in generally challenging ecological niches indicates a high degree of physiological resilience. In this respect, examining environmental microorganism samples and evaluating them for antibiotic resistance becomes important.

*Salmo munzuricus*, as its name suggests, is an endangered trout species first identified in the Muzur River, but later found in other areas of the Euphrates basin (Turan et al. 2017; Turan et al. 2021). It is closely related to other regional Salmo species, but can be distinguished by its size and specific fin and body proportions, as well as increased, irregularly shaped black spots (more than 80 in adults). Genetic studies confirm its close relationship and uniqueness with *Salmo baliki* (Turan et al. 2021). In addition to its cultivation and cultivability, its reproductive and spotting characteristics, stomach contents, and feeding habits, nutrient content, heavy metal accumulation, and anesthetic applications have been studied by various researchers (Can et al. 2022). None of the studies conducted on this species has examined the antimicrobial resistance (AMR) dynamics or the role of this species as a potential resistance reservoir. It is known that factors such as antibiotic use in aquaculture, agricultural runoff, and wastewater discharge contribute to the emergence and spread of AMR in natural waters (Alem et al. 2025). These environmental pressures can create a selective environment suitable for opportunistic pathogens to acquire resistance, especially in sensitive and endemic species (Bengtsson‐Palme et al. 2018). *Kocuria rhizophila* and *Micrococcus luteus* (Pękala et al. 2018), recently identified as opportunistic pathogens in species such as brown trout (*Salmo trutta* Linnaeus 1758) and rainbow trout (*Oncorhynchus mykiss* Walbaum 1792), do not yet have a fully understood status in fish species living in their natural habitat. There are very few studies on *Kocuria* sp. and *Micrococcus* sp. These studies have not gone beyond their isolation from farmed salmon. Furthermore, none of these studies contains information about the fish's health status or the type of bacteria (Pękala et al. 2018). This situation, especially in wild and endangered species, constitutes an important research gap regarding the potential role in the spread of AMR in bacteria such as *Kocuria rhizophila*. Considering the indispensable role of fish in various ecological niches and their prevalence in aquatic environments, the primary aim of this study is to isolate the bacterium Kocuria rhizophila in wild mountain trout (*Salmo munzuricus*) samples and to verify its presence in the natural habitat of this species by characterizing it molecularly. The second and more important aim of the study is to determine the antimicrobial resistance profile of the isolated bacterium against clinically important antibiotics, thereby revealing the potential of this endemic species as an indicator species in terms of AMR, and providing preliminary data on the environmental resistance burden in the region. In this way, it is aimed to shed light on both the conservation of the species and the current state of the AMR issue—important in terms of public health—in wild fish populations.

## 2 Materials and Methods

### 2.1 Ethics Approval

All experimental procedures were carried out in accordance with the guidelines established by Munzur University (Approval No:2022/22‐06/15‐02). In addition, the study was conducted in accordance with ARRIVE guidelines.

### 2.2 Study Area and Fish Sampling

The study was carried out in the Munzur River (Tunceli, Türkiye), which is an important freshwater ecosystem known for its cold water sources. A total of 30 wild *Salmo munzuricus* specimens were collected alive using fish capture cages between November and December 2024. Sampling was conducted from two separate locations with different environmental characteristics (Ovacık‑39°09'59.5“N/39°27'49.1“E and Koyungolu‑39°20'50.8“N/39°08'06.0“E). Although both regions are relatively unpolluted, they are known to be exposed to effects originating from nearby agricultural activities and local domestic waste. This situation was thought to potentially affect the microbiological profile of the aquatic environment.

### 2.3 Morphological and Molecular Characterization of Fish Specimens

**Figure 1:**
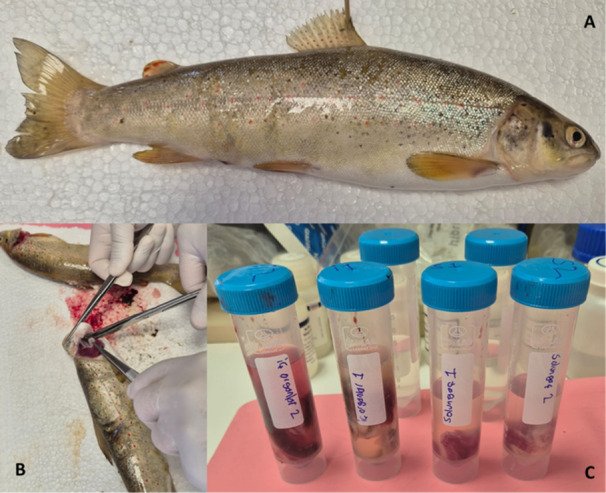
Fish specimen used in the study (A), sampling from gills for bacterial isolation (B), and transfer of gill and digestive organ samples to physiological saline (C).

Morphological and molecular identification was performed to determine that the collected fish samples belonged to the species *Salmo munzuricus*. Morphological identification was performed using the species key proposed by Küçük et al. (2024) (Figure 1‐A), while mitochondrial cytochrome b gene sequences were used for molecular identification. DNA was isolated from all collected fish samples. DNA isolation was performed in two ways using the adipose fins in the samples. Either the DNeasy Tissue Kit (QIAGEN) was used, following the manufacturer's protocol, or the phenol‐chloroform extraction method was employed (Sambrook et al. 1989). A Nanodrop 2000 C spectrophotometer (Thermo Scientific, Waltham, MA, USA) was used to determine the purity and concentration of the obtained DNA samples. DNA samples with the desired purity were stored at ‐20°C for use in PCR testing. For PCR procedures, forward and reverse primers (F:GCTAATGACGCACTAGTCGACC‐R: CAGGTGAGGATAAGTATGTCTGCTAC) (Kar 2006) were used that amplified a region of approximately 971 bp for the Cyt‐b region. PCR reactions were performed in a total volume of 40 µL containing 600 ng genomic DNA, 2.5 U Taq DNA polymerase, 5 µL of 10 X Taq buffer (100 mM Tris‐HCl, 500 mM KCl, pH 8.3), 200 µM dNTP, and 10 pmol of each primer. For amplification, the protocol in the instrument (BIO‐RAD) consisted of an initial denaturation at 95°C for 5 min, followed by 35 cycles of denaturation at 94°C for 1 min, annealing at 68°C for 1 min, extension at 72°C for 1 min, and finally a final extension at 72°C for 5 min. To check the quality of the amplification products, electrophoresis was performed on a 1.5% agarose gel containing ethidium bromide. Positive PCR products were sent to Iontek Laboratories (Istanbul, Turkey), a commercial company, for Sanger sequencing using the BigDye Terminator v3.1 Cycle Sequencing Kit (Applied Biosystems, Life Technologies Corporation, Austin, TX, USA). Sequencing was performed bidirectionally using primers for amplification of the analyzed mitochondrial genome regions. Raw array data was visualized and processed using ChromasPro V1.34 and Clone Manager 10, respectively. Phylogenetic trees were used to more easily express and visualize the molecular identification of the obtained fish. This was done based on cyt‐b sequences obtained from the fish studied and reference data from NCBI GenBank for Salmo genus representatives distributed in Türkiye found in the database. In order to compare the data obtained in the study with local sources, phylogenetic relationships between species were analyzed using MEGA X (Kumar et al. 2018), and Bayesian inference (BI) analysis was performed using MrBayes 3.2 software (Ronquist et al. 2012) as suggested by Küçük et al. (2024). To find the root of the generated phylogenetic trees, sequence data from *Salmo salar* (JX960834.1) were identified as outgroups. Branches with 50% or more bootstrap support were considered significantly supported (Shen et al. 2011). Phylogenetic trees obtained using Cyt‐b sequences were visualized using the Interactive Tree of Life (iTOL) web server (http://itol.embl.de/) (Letunic and Bork 2019).

### 2.4 Isolation and Morphological Analysis of Bacterial Samples

Fish samples from which bacteria were to be isolated were caught alive from the water and transported directly to the laboratory within a maximum of 2 h. The sampled fish did not show any apparent clinical signs of disease. No external or internal lesions were observed during examination. Fish samples were first rinsed under running water to remove physical residues and surface mucus, and then washed a second time with sterile pure water. Afterwards, the samples were placed into separate sterile bags in a way that prevents them from coming into contact with each other. Tissue samples were taken from the gills and digestive systems of dying fish using aseptic scalpels and tweezers for bacteriological examination. The samples were then diluted in sterile physiological saline solution at a 1:1 (w/v) ratio and homogenized (Pękala et al. 2018). The resulting solutions were surface‐inoculated onto Tryptic Soy Agar (TSA‐Merck). The resulting petri dishes were incubated at 27°C and 37°C for 3–5 days. These two temperature values were selected to determine the presence of both fish‑associated/environmental bacteria and bacteria that may have potential importance for warm‑blooded hosts. Samples with dense growth in Petri dishes were inoculated with Tryptic Soy Broth (TSB‐Merck), passaged, and the dominant bacterial colony types were re‐isolated and incubated at 27°C and 37°C for 24–48 h. Samples were separated according to different morphological, biochemical, and Gram‐negative characteristics. The prevalence of yellow colonies was considered, and these colonies were used for further analysis. Since TSA is a non‐differentiating medium, the yellow color was evaluated not as a metabolic reaction but as a result of natural pigment production produced by the isolates. Pure cultures were re‐cultured in liquid culture and frozen at −80°C using 15% glycerol (v:v) for further study.

### 2.5 Identification of Bacterial Samples by MALDI‐TOF MS

Considering the temperatures selected inside the isolation, it was seen that the yellow‐colored colonies developed successfully at 27°C in terms of both faster reproduction and prevalence. In this respect, yellow colonies isolated from fish taken from the stock were removed from the stock and incubated on agar medium (TSA) at 25°C ± 2°C for 18 to 24 h before testing. Single and pure colonies obtained were prepared for MALDI‐TOF MS as previously described (Sogawa et al. 2011). Briefly, a single colony was placed directly onto an MTP BigAnchorChip 384 TF target plate (Bruker Daltonics), and afterward, 1 μL of saturated α‐CHCA matrix solution prepared in 50% acetonitrile–2.5% trifluoroacetic acid was added onto it and air‐dried at room temperature to ensure crystallization. Afterwards, species‐level identification was performed using the MALDI Biotyper CA System (Bruker Daltonics, Billerica, MA). Identification services were provided through a service contract with the Hatay Mustafa Kemal University Plant Health Application and Research Center.

### 2.6 16S rDNA PCR Analysis and Molecular Identification of Bacterial Samples

Two isolates collected from *Salmo munzuricus* trout were used for 16S rDNA gene sequencing. Total genomic bacterial DNA was extracted using the Bacterial Genomic DNA Isolation Kit (Hibrigen) following the manufacturer's instructions. A Nanodrop 2000 C spectrophotometer (Thermo Scientific, Waltham, MA, USA) was used to determine the purity and concentration of the obtained DNA samples. Suitable DNA samples were amplified using the primers 27 F (5′‐AGAGTTTGATCCTGGCTCAG‐3′) and 1492 R (5′‐TACGGCTACCTTGTTACGACTT‐3′) (Weisburg et al. 1991), which are used for the universal 16S rRNA gene region, to obtain regions of approximately 1495 bp in length for use in the PCR process. The PCR reaction was carried out in accordance with the study by Bektas et al. (2024). After separation of the amplification products on a 1.5% (w/v) agar gel in 1X TBE buffer, ethidium bromide staining and UV imaging were performed. DNA sequencing of PCR products was performed bidirectionally using the Sanger sequencing method with the help of a 3130xl Genetic Analyzer (Applied Biosystems, USA). The sequencing process was provided through a service procurement contract at the Kahramanmaraş Sütçü İmam University University‐Public‐Industry Cooperation Development Application and Research Center. 16S rDNA sequence data were determined using the Basic Local Alignment Search Tool (BLAST) analysis at the National Center for Biotechnology Information (NCBI), paying attention to a 90%–100% similarity rate at the species level.

### 2.7 Antimicrobial Susceptibility Test

The antimicrobial susceptibility of *Kocuria rhizophila* MGLB‐20 and MGLB‐21 strains, which were morphologically and molecularly characterized and stockpiled, was tested according to the Kirby‐Bauer antibiotic test method. The disk diffusion method was performed on Mueller‐Hinton agar (Merck). While a blank disk was used as a negative control, the susceptibility of the tested organisms was determined using 11 different antibiotics, including Amoxiillin (AMX25‐25 μg), Ampicillin (AM10‐10 μg), Chloramphenicol (C30‐30 μg), Ciprofloxacin (CIP5‐5 μg), Erythromycin (E15‐15 μg), Gentamicin (CN10‐10 μg), Penicillin G (P10‐10 μg), Rifampin (RA5‐5 μg), Streptomycin (S10‐10 μg), Tetracycline (TE30‐30 μg), and Vancomycin (VA30‐30 μg), for susceptibility testing. 100 μl overnight cultures of each indicator pathogen sample used in the study were used after being adjusted to 1–1.5 × 10^8^ CFU mL^−1^ according to the McFarland 0.5 standard. After inoculation, solid media were incubated at 27°C for 24 h, and the diameters of the inhibition zones were measured to estimate the antimicrobial susceptibility of the collected isolates. Tests were performed in triplicate. The obtained results were evaluated by comparing them as susceptible, moderately susceptible, resistant according to the breakpoint values specified in the CLSI (or EUCAST) standards. In addition, because CLSI interpretation criteria are not available for *Kocuria*, the antimicrobial susceptibility test results of the *Kocuria* isolates were interpreted by taking *Staphylococcus* and *Enterococcus* as reference.

### 2.8 Statistical Calculations

GraphPad Prism 8 software was used for statistical analysis. All tests were performed in triplicate, and values were expressed as mean ± standard errors of the mean. Results were evaluated using analysis of variance (ANOVA) with Duncan Multiple Comparison or least significant difference (LSD) tests at *p* ≤ 0.05 and *p* ≤ 0.01 (Steel and Torrie 1980; Torğut et al. 2022).

## 3 Results

### 3.1 Morphological and Molecular Characterization

A morphological characterization analysis was conducted on 30 *Salmo* sp. individuals. In morphometric analyses, nine basic criteria were used for species identification and for determining phenotypic variation among populations. While standard length (SL), body depth (BD), and anterior dorsal distance (AD) characterize the general body form; head length (HL), head depth (HD), eye diameter (ED), and snout length (NL) revealed distinctive features related to head morphology. Dorsal fin length (DFL) and caudal peduncle depth (CPD) were evaluated as important morphological indicators supporting taxonomic differences in fin and locomotor structure (data is not provided here). Of the fish samples used in the study, 27 were identified as *Salmo munzuricus*, morphologically matching the species key proposed by Küçük et al. (2024). However, DNA was obtained from all *Salmo* sp. samples collected for molecular identification and used in molecular analyses.

**Figure 2:**
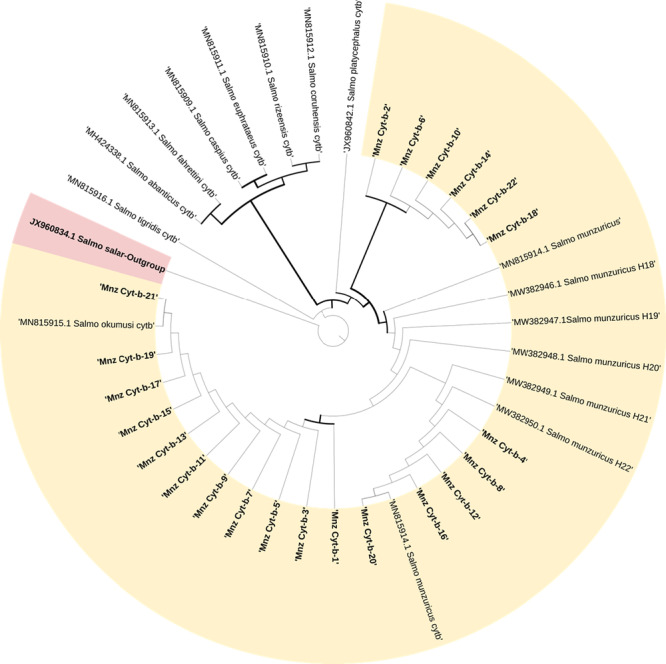
Fish samples with molecular identification based on the Cyt‐B gene region; yellow indicates the *Salmo munzuricus* branch, red indicates the outgroup sample, and dark lines indicate the main branches.

Cyt‐b gene barcode sequence amplification was performed in all samples, yielding a band of approximately 971 bp in all samples. The amplification products were sequenced, and the resulting sequences were aligned to a consensus length of 971 bp. BLASTn analysis performed on the NCBI database confirmed that all samples were identified as *Salmo munzuricus* with values ranging from 97% to 100%. Phylogenetic analysis of all Cyt‐b sequences obtained in this study was performed using the NJ method and *Salmo salar* (Ac. no. JX960834.1) as an outgroup. To better explain the molecular characterization of the obtained fish samples, a phylogenetic tree was constructed using genetic distance values, as shown in Figure 2. Examination of the tree clearly shows that the fish samples obtained were found in the same clay as *Salmo munzuricus* (MN815914) and its haplotypes previously identified in Türkiye, indicating that the samples in this study are pure Salmo munzuricus samples.

### 3.2 Bacteria Isolation

**Figure 3:**
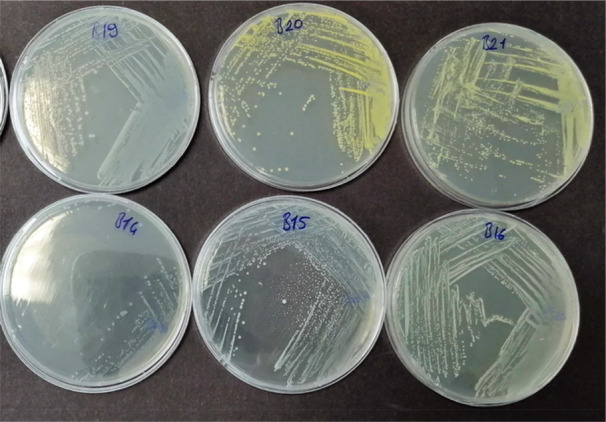
Some of the isolated and purified bacteria.

Growth was observed at both of the temperatures used for bacterial isolation (37°C and 27°C). However, while more pronounced growth was detected at 27°C, more limited growth was determined at 37°C. In addition, one hundred different bacterial sample were identified from the gills and digestive organs of *Salmo munzuricus* specimens based on their morphological, biochemical, and Gram‐negative characteristics. In the initial observations, it was determined that gram positive bacteria were dominant in the population. Of the isolated bacteria, 42% were obtained from the gills, and 58% were obtained from the intestines. In addition, it was determined that 53% of the bacteria formed yellow‐colored colonies. However, considering the clear morphological difference in yellow coloration compared to other bacterial samples and its prevalence, the study was continued using these colonies (Figure 3). In other words, the predominant growth of this colony type in the isolation sources led us to consider these bacteria. Yellow bacterial colonies, approximately 2‐3 mm in diameter, were identified under a microscope as Gram‐positive cocci arranged in tetrads. Before proceeding to the molecular identification and antibiotic resistance analysis of these bacteria, a preliminary screening was carried out with three antibiotics (Penicillin G, Ciprofloxacin, and Tetracycline). Since Penicillin G is effective against gram‑positive bacteria, Ciprofloxacin was selected for the evaluation of the general resistance profile; and Tetracycline was selected for the examination of the widespread resistance profile. The isolates showing the highest resistance to these three antibiotics were identified and used in subsequent analyses. In this regard, among the yellow colonies originating from the gills, MGLB 20; and among the intestinal flora isolates, MGLB 21 showed higher antibiotic resistance compared to the others.

Bacterial isolates identified with different codes, such as MGLB 1‐100, were subjected to polyphasic identification combining proteomic fingerprinting and genotyping. In the initial identification using MALDI‐TOF, a high‐reliability spectral log (score) was obtained (N > 2.00), and further studies were conducted on the samples (MGLB‐20 and MGLB‐21). MALDI‐TOF values showed that both isolated species had highly probable species identification (Score Value: 2.300–3.000). This value significantly exceeded the reference Kocuria rhizophila spectrum value in the identification device used (Bruker Biotyper DB). To validate this proteomic profile, the 16S rRNA gene was amplified and sequenced (~ 1495 bp). The obtained sequence results were identified as Kocuria rhizophila with 99.51% nucleotide identity (E value: 0.0) in the BLASTn homology performed in the NCBI GenBank database. The similarity between the high MALDI‐TOF score and the 16S rDNA sequence homology (> 98.7% species limit) proved that the obtained isolations were most likely Kocuria rhizophila. Antimicrobial resistance values were evaluated using two strains with MALDI‐TOF and 16S rDNA sequence blast results above 99% (Table 1).

**Table 1:** MALDI‐TOF and 16 s rDNA sequencing analysis results.

Isolation Code	Organism (best match)	MALDI‐TOF MS	NCBI
		Score Value	Blast Score (%)
MGLB‐20 (Ovacik)	*Kocuria rhizophila*	2.133‐2.147 (Taxonomy ID: 72000)	99.72% (Ac. No. MH715218.1)
MGLB‐21 (Koyungolu)	*Kocuria rhizophila*	2.142‐2.374 (Taxonomy ID: 72000)	99.51% (Ac. No. MK312487.1)

Considering the 16S rDNA region in both isolated strains, the similarity of over 99.72% between *Kocuria rhizophila* MGLB‐20 and strain MH715218.1 isolated from hot spring sediments was interpreted as indicating that this bacterium migrated from the hot springs in the region to the *Salmo munzuricus* species. Regarding other strains, the similarity between *Kocuria rhizophila* MGLB‐21 and MK312487.1, which is considered to be 99.51% insect‐based, is seen as a result of meeting the nutritional needs of the fish in their natural environment. Regardless of the method, the presence of this bacterium in a wild fish species living in its natural environment has the potential to cause several problems. From this perspective, the spread of antibiotic resistance in wild fish poses significant risks to both the environment and human health. It is estimated that these bacteria somehow passed from the soil to the water and then to the fish. In this respect, it can be said that their acquisition of resistance characteristics is of external origin.

### 3.3 Antibiotic Resistance Profile of Isolated Bacteria

**Figure 4:**
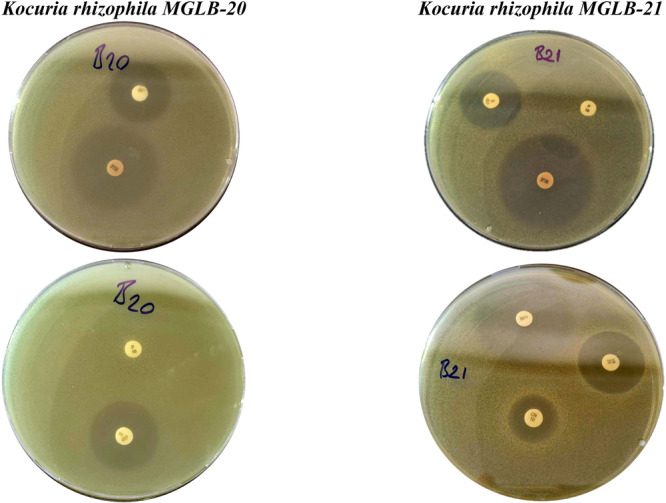
Antimicrobial susceptibility of *Kocuria rhizophila* isolates.

When the inhibition data obtained are examined, it is shown that *Kocuria rhizophila* isolates (MGLB‐20 and MGLB‐21) obtained from *Salmo munzuricus* may have a gradual resistance profile. When the values presented in Table 2 are examined, significant differences were observed among standard pathogenic microorganisms against different antibiotics such as Penicillin G, Gentamicin, Streptomycin, and Tetracycline (*p* < 0.01). The most striking finding in the data obtained in the study is that the *Kocuria rhizophila* isolates (MGLB‐20 and MGLB‐21) show complete resistance to Penicillin G, whereas the reference pathogenic strains remain sensitive to this antibiotic (Figure 4). In order to verify the effectiveness of the experimental setup and the antibiotic disks, quality control strains were selected in accordance with CLSI standards; within this scope, the fact that the *Enterococcus faecalis ATCC 29212* and *Staphylococcus aureus ATCC 25923* strains used produced results within the expected sensitivity range confirms the accuracy of the resistance data on the isolates. The reason for choosing the strains examined in the study to be gram positive is that it allows comparison with clinically and veterininarily important pathogens. However, both MGLB‐20 and MGLB‐21 strains showed moderate susceptibility to Gentamicin, Streptomycin, and Tetracycline. In contrast, the isolates showed low resistance (high susceptibility) to the majority of the spectrum, including Amoxicillin, Rifampicin, Ampicillin, Chloramphenicol, and Erythromycin.

**Table 2:** Comparison of antimicrobial resistance properties of pathogenic microorganisms and *Kocuria rhizophila* isolated from Salmo munzuricus fish in the natural environment, according to their inhibition zones (mm±SD).

Standard Antibiotics	Test Microorganisms				CLSI Microorganisms		
	*Kocuria rhizophila MGLB‐20*	*Kocuria rhizophila MGLB‐21*	*Enterococcus faecalis (ATCC 29212)*	*Staphylococcus aureus subsp. aureus (ATCC 25923)*	*Enterococcus spp*. (S/I/R)***	*Staphylococcus spp*. (S/I/R)***	P
***Amoxicillin (25 μg)***	40.2 ± 7.58	36.53 ± 7.36	ND	ND	≥ 17/‐/ ≤ 16	—	*
***Ampicillin (10 μg)***	32 ± 0.4	28.9 ± 1.65	**0.0** ± **0.0** ^**a**^	**0.0** ± **0.0** ^**a**^	≥ 17/‐/ ≤ 16	—	**
***Chloramphenicol (30 μg)***	32.8 ± 3.73	31.16 ± 2.28	26.33 ± 1.45	19.26 ± 0.40	≥ 18/13‐17/ ≤ 12	≥ 18/13‐17/ ≤ 12	**
***Ciprofloxacin (5 μg)***	23.46 ± 0.65	23.76 ± 2.05	ND	ND	≥ 21/16‐20/ ≤ 15	≥ 21/16‐20/ ≤ 15	*
***Erythromycin (15 μg)***	31.16 ± 1.85	28.7 ± 1.81	18.06 ± 0.45	17.2 ± 0.79	≥ 23/14‐22/ ≤ 13	≥ 23/14‐22/ ≤ 13	**
***Gentamicin (10 μg)***	**12.26** ± **1.41** ^**b**^	**12.36** ± **1.30** ^**b**^	**13.9** ± **1.73** ^**b**^	**13.3** ± **0.60** ^**b**^	—	≥ 15/13‐14/ ≤ 12	**
***Penicillin G (10 μg)***	**0.0** ± **0.0** ^**a**^	**0.0** ± **0.0** ^**a**^	20.16 ± 1.28	21.06 ± 0.75	≥ 15/‐/ ≤ 14	≥ 29/‐/ ≤ 28	**
***Rifampin (5 μg)***	36.6 ± 0.4	37.43 ± 0.87	12.86 ± 1.04	14.63 ± 0.83	—	≥ 20/17‐19/ ≤ 16	**
***Streptomycin (10 μg)***	**17.7** ± **0.98**	**18.93** ± **0.89**	19.63 ± 1.13	20.3 ± 0.62	—	—	**
***Tetracycline (30 μg)***	**28.56** ± **2.60**	**28.7** ± **3.98**	33.96 ± 2.75	29.33 ± 3.34	≥ 19/15‐18/ ≤ 14	≥ 19/15‐18/ ≤ 14	**
***Vancomycin (30 μg)***	26.26 ± 1.20	21.93 ± 0.97	20.8 ± 1.25	19.5 ± 1.25	≥ 17/15‐16/ ≤ 14	—	**

*Note:* The data are presented as the means ± standard deviation (SD) of three independent samples; ND means not detected. Amoxicillin (AMX25‐25 μg), Ampicillin (AM10‐10 μg), Chloramphenicol (C30‐30 μg), Ciprofloxacin (CIP5‐5 μg), Erythromycin (E15‐15 μg), Gentamicin (CN10‐10 μg), Penicillin (P10‐10 μg), Rifampin (RA5‐5 μg), Streptomycin (S10‐10 μg), Tetracycline (TE30‐30 μg) and Vancomycin (VA30‐30 μg), ^a‐b^Smallest zone diameter. ^a^:It means that no measurable inhibition zone has formed. Significant effect; * *p* < 0.05, ** *p* < 0.01. ***S: Sensitive, I: Moderately Sensitive, R: Resistant.

## 4 Discussion

Türkiye is an important center for *Salmo* diversity, hosting many different fish species from the Black Sea to the Mediterranean. Initial studies reported only a limited number of *Salmo* species in this region, including *S. abanticus*, *S. caspius*, and *S. platycephalus* (Turan et al. 2011). However, recent systematic research has led to a significant expansion of this knowledge. To date, at least 10 different *Salmo* species have been identified in Anatolia, including *S. rizeensis, S. coruhensis, S. tigridis, S. labecula, S. opimus, S. chilo, S. kottelati, S. okumusi, S. euphrataeus*, and *S. munzuricus* (Turan et al. 2014; Turan et al. 2021). These recently identified species highlight that Türkiye's ichthyological diversity is still not fully documented. In this study, the molecular identity of *Salmo munzuricus*, which is one of these species, was verified by Cyt‐b gene sequencing (971 bp) and phylogenetic analysis; high sequence identity (97%–100%) and clustering with previously reported haplotypes showed that the sampled fish represent genetically pure populations. This finding constitutes a critical basis for understanding the microbial colonization and resistance dynamics of host‐specific ecological conditions. Furthermore, there are very few studies on the microflora of these fish specimens.

The ability of *K. rhizophila* to metabolize plant material is crucial; this is highly advantageous for its survival and reproduction in environments rich in decaying organic matter, such as aquatic systems (Takarada et al. 2008). The ability to thrive in a wide variety of, often challenging, ecological niches indicates a high degree of physiological resilience. In this respect, examining environmental samples and evaluating them in terms of antibiotic resistance becomes important. The fact that *Kocuria rhizophila* was not previously identified as a fish pathogen, and its recent appearance as a disease agent in salmon (Pękala et al. 2018), presents it as a “newly emerging disease” or “newly emerging pathogen” in the field of fish pathology. This bacterial species, previously isolated from farm‐raised trout, was isolated for the first time in this study from the naturally occurring Salmo munzuricus species in the Tunceli region, and its antimicrobial properties were investigated. The bacterial isolation results revealed the dominance of Gram‐positive microorganisms; in particular, a marked abundance of yellow‑pigmented colonies was observed, and these were later identified as *K. rhizophila*. Higher bacterial growth at 27°C compared to 37°C suggests environmental adaptation to ambient water temperatures and strengthens the ecological origin of these isolates. Urgent and detailed research is needed to elucidate the specific diet, precise reproductive biology (including spawning sites, timing, and environmental conditions), migration patterns, and complete life cycle of *Salmo munzuricus*. Furthermore, there are no studies on the species‐specific microflora.

In this study, the antimicrobial susceptibility profiles of the *Kocuria rhizophila* isolates (MGLB‐20 and MGLB‐21) obtained from *Salmo munzuricus* were examined, and they were found to have a moderate level of antibiotic resistance. The polyphasic identification approach (MALDI‑TOF proteomic profiling and 16S rRNA gene sequencing) confirmed that both isolates are *K. rhizophila*, with high MALDI‑TOF scores (2.300–3.000) and more than 99% sequence homology. Phylogenetic analyses indicate different ecological origins of the isolates: while MGLB‑20 appears to be closely related to strains from thermal spring sediments, MGLB‑21 shows similarity to insect‑associated strains. These findings support the hypothesis that environmental transmission routes, such as soil‑water‑fish or diet‑mediated transfer, play a critical role in shaping the microbiota of *S. munzuricus*. Overall, the data indicate that while Kocuria, which can be isolated from fish living in natural environments, may have a benign resistance profile, the development of resistance to Penicillin, Gentamicin, Streptomycin, and Tetracycline suggests it may also be susceptible to other antibiotics. This has been interpreted as being due to their initial isolation from the natural environment, as well as their lower exposure to antibiotics compared to other bacteria that are resistant to antibiotics.

*Kocuria rhizophila* strains (MGLB‐20 and MGLB‐21) isolated from Munzur trout were found to be resistant to the standard Penicillin G disc, while it is noteworthy that the same isolates showed high susceptibility to Amoxicillin and Ampicillin, synthetic Penicillins structurally similar to Penicillin G. In this study, the inhibition zones for amoxicillin were measured as 40.2 ± 7.58 mm and 36.53 ± 7.36 mm; and for ampicillin, they were measured as 32 ± 0.4 mm and 28.9 ± 1.65 mm, and these values exceed or are consistent with the Clinical and Laboratory Standards Institute (CLSI) susceptibility thresholds. This situation is the exact opposite in standard strains. Indeed, the reference strains used, *Enterococcus faecalis* ATCC 29212 and *Staphylococcus aureus* ATCC 25923, showed complete resistance (0.0 mm) to ampicillin, while exhibiting susceptibility to penicillin G (20.16 ± 1.28 mm and 21.06 ± 0.75 mm, respectively). This contrast emphasizes that the resistance load in environmental isolates is potentially lower compared to clinical strains and has been interpreted as the presence of species specific resistance mechanisms. This situation is the opposite in standard strains. In this respect, the fact that the isolates in this study are resistant only to natural Penicillin G but highly susceptible to synthetic aminopenicillins points to the existence of a specific microbiological mechanism. Penicillin G is a β‐lactam antibiotic that disrupts cell wall synthesis by inhibiting the third step (transpeptidation and carboxypeptidation) of peptidoglycan synthesis (Bush and Bradford 2016). Beta‐lactam antibiotics inhibit the final step of peptidoglycan synthesis by acylating transpeptidase, which plays a role in the cross‐linking of peptides to form peptidoglycans. The targets of beta‐lactam antibiotics are known as penicillin‐binding proteins (PBPs) (Bush and Bradford 2016). This binding disrupts the terminal transpeptidation process and leads to loss of viability and lysis through autolytic processes within the bacterial cell (Eckburg et al. 2019).

In this study, the antimicrobial susceptibility profiles of the *Kocuria rhizophila* strains (MGLB‐20 and MGLB‐21) isolated from *Salmo munzuricus* revealed that the isolates generally showed high susceptibility to antibiotics, but exhibited moderate resistance to some antibiotics. In particular, complete resistance to penicillin G (0.0 mm inhibition zone) was observed, while low inhibition zones for gentamicin (~12 mm) suggest reduced susceptibility or moderate resistance when compared with CLSI breakpoints for *Staphylococcus* species. In contrast, the isolates exhibited high susceptibility to a wide range of antibiotics, including amoxicillin, ampicillin, chloramphenicol, erythromycin, and rifampin. The erythromycin and ciprofloxacin results also support this finding. In addition, the measured inhibition zones are mostly above the CLSI susceptibility thresholds. The statistically significant differences observed in the antibiotics used in the study (*p* < 0.01) strengthen the reliability of these patterns and indicate that the antimicrobial responses did not result from random variation. Salmon fish host a wide variety of bacteria that can affect disease dynamics in populations. In studies conducted, it has been determined that the most common Gram‐negative bacteria originating from salmon are Vibrio spp. and Aeromonas spp. (Barnes et al. 2022). Especially species such as A. salmonicida, A. hydrophila, and A. sobria play an important role as both obligatory and opportunistic pathogens in freshwater fish (Wiström et al. 1998; Králová et al. 2016). It is known that these species can turn into opportunistic pathogens under environmental stress conditions and form infections (Austin and Austin 2007). In addition, it has been reported that Aeromonas species can exhibit natural resistance to penicillin‐group antibiotics due to chromosomal beta‐lactamases expressed at low temperatures (Akinbowale et al. 2006). In their study, Marijani (2022) stated that a small percentage of some Salmonella species isolated from fish were resistant to gentamicin, tetracycline, penicillin, and erythromycin. Similarly, Ponce et al. (2008) found that most of the isolates were resistant to Ampicillin, Tetracycline, Chloramphenicol, Gentamicin, Sulfisoxazole, Streptomycin, and Kanamycin. In this study, however, the isolates’ high susceptibility to ampicillin and chloramphenicol once again demonstrates that the resistance profiles of environmental strains may differ from clinical isolates and that the development of resistance is closely related to the level of antibiotic exposure (Aga et al. 2025). Oates et al. (2012) reported that the transport of agricultural waste into freshwater and saltwater pathways can cause resistant bacteria to cause diseases in organisms in both freshwater and saltwater, and can contribute to antibiotic resistance. Another effect is the increased prevalence of antibiotic resistance genes in aquatic environments, increasing the risk of resistant bacteria infecting animals or their surroundings. Animals may become ill, or resistance traits may be transferred to other bacterial species, or aquatic environments may become a reservoir for the transfer of bacteria and resistance back to humans and the environment (Greig et al. 2007; Schaefer et al. 2009). Natural aquatic environments can serve as reservoirs for antibiotic‐resistant pathogens (Preena et al. 2020). The physicochemical parameters of water also influence the increased antibiotic sensitivity among aquatic bacteria. The selection of antibiotic‐resistant organisms in natural aquatic environments primarily results from the natural production of antibiotics within the system, waste products from antibiotic‐treated animals/humans, terrestrial runoff, inputs from animal feed and crops, waste discharges, and domestic sewage containing antibiotics (Kumar et al. 2017). Specifically, sewage waste has been shown to have an impact on antibiotic‐resistant enteric pathogens in wild marine species, as confirmed by comparing fish from polluted and unpolluted areas (Al‐Bahry et al. 2009). This is why the introduction of different antibiotics into natural environments can create selective pressure that promotes the emergence and spread of antibiotic resistance in aquatic organisms. In this study, the resistance shown by the *K. rhizophila* strains isolated from *S. munzuricus*, particularly to antibiotics such as penicillin G and gentamicin, may indicate the presence of these antibiotics at low levels in natural aquatic environments. However, the isolates’ high susceptibility to a wide range of antibiotics led to the conclusion that widespread multidrug resistance has not yet developed in this population and that the resistance load is limited. In a study examining high and low anthropogenic activity, wild freshwater species in reservoirs with higher anthropogenic activity showed resistance genes to sulfonamide, quinolone, and erythromycin, while those in reservoirs with lower anthropogenic activity showed only beta‐lactam resistance genes (Marti et al. 2018). In accordance with these findings, the fact that the isolates in this study were resistant to penicillin G from the beta‑lactam group of antibiotics but susceptible to other beta‑lactams may be an indication of a selective pressure reflecting the low anthropogenic activity in the sampling area. Thus, they determined the effect of recreational activities on the formation of AMR genes in the natural aquatic environment. This means that antibiotic‐resistant wild fish can be taken as a bioindicator of pollution in natural marine and freshwater environments (Preena et al. 2020). The findings of this study also suggest that the antibiotic resistance profiles detected in an endemic freshwater species such as *S. munzuricus* may be used as a sensitive biomarker of environmental pollution and antimicrobial influence in its habitat.

The use of antimicrobials has the potential for AMR growth among microorganisms. It is important to acknowledge that antimicrobials are frequently used worldwide for prophylactic purposes and as growth accelerators in livestock and agriculture. Given that humans and animals regularly exchange resistant bacterial strains within the ecosystems in which they live together, the development of AMR is inevitable (Milijasevic et al. 2024). The most commonly detected substances in the fish were Tetracyclines, β‐lactams (Penicillin), and Phenicols (Chloramphenicol) (Moffo et al. 2024). Particular attention should be paid to antimicrobial agents such as Chloramphenicol and Tetracycline, which are toxic to humans even at low concentrations (Moffo et al. 2024). In this study, the isolates’ high susceptibility to chloramphenicol and tetracycline (together with moderate inhibition zones for tetracycline) indicates that resistance to these agents has not yet been selected and that environmental pressure is limited. It is a known fact that antibiotics such as Tetracycline, Oxytetracycline, Penicillin, Erythromycin, Enrofloxacin, and Sulfamethoxazole are used in fish farms. These antimicrobial agents, classified by the World Health Organization as high‐priority critical antimicrobial agents or very important antimicrobial agents, underscore the urgent need for antimicrobial regulation, strengthening, control, and reporting in aquaculture (Moffo et al. 2024). It can be said that the resistance profiles of the *Kocuria rhizophila* isolates in the study are an effective indicator of the low level pollution and antimicrobial exposure in the region. When AMR in environmental microorganisms is evaluated in terms of the WHO AWaRe classification, the fact that sensitivity to ampicillin and amoxicillin is preserved while full resistance is shown to penicillin G demonstrates to us that there is a species specific beta lactam resistance mechanism shaped by the environment rather than clinical exposure (Singer et al. 2016). Medium level resistance to gentamicin and streptomycin, on the other hand, indicates the presence of ongoing selective pressure in aquatic ecosystems (Okeke et al. 2022). In contrast, the absence of resistance detected to antibiotics in the Reserve group suggests that the isolates have not yet acquired high risk clinical resistance mechanisms, but this does not mean that there is no potential danger. The environmental resilience of *K. rhizophila* and the prevalence of horizontal gene transfer in aquatic environments show that such isolates may indirectly affect human, animal, and environmental health by acting as a reservoir for resistance genes (Pleshko and Zhurina 2025). The findings can be said to indicate that monitoring wild endemic species with global standards such as AWaRe and protecting natural habitats are critical for understanding the spread of environmental resistance and reducing global AMR risk (Zhang et al. 2024). Effective monitoring of environmental antimicrobial resistance is crucial for assessing the risk of AMR contamination to human and animal health (Tskhay et al. 2025). Recent studies indicate that the overuse and misuse of antibiotics mean a global antimicrobial resistance crisis is on the horizon (Tskhay et al. 2025). With approximately five million deaths linked to resistant bacteria each year, AMR has become one of the greatest threats to human health (Tskhay et al. 2025). Addressing this global AMR crisis requires a comprehensive strategy that integrates human health, animal health, and the environment (Tskhay et al. 2025). However, despite the significant contribution of the environment to the emergence and spread of AMR, current surveillance strategies primarily focus on clinical, food, and agricultural settings (Tskhay et al. 2025).

## 5 Conclusion

Endemic species like *Salmo munzuricus* are among the greatest evidence of Türkiye's rich, yet undiscovered biodiversity. Although some characteristics of such species have been revealed, there are no studies on the species‐specific microflora. This study has revealed for the first time the isolation of *Kocuria rhizophila* from *Salmo munzuricus* and has identified the isolates' unique antimicrobial susceptibility profiles. The *Kocuria rhizophila* MGLB‑20 and MGLB‑21 strains showed distinct resistance to penicillin G and gentamicin, but by remaining generally susceptible to other antibiotics, they exhibited a specific and limited resistance profile. The findings show that the fish's microbiota is shaped by environmental interactions and that there is low antimicrobial exposure in natural waters. The presence of resistant bacteria in an endemic species emphasizes that natural ecosystems may be a potential reservoir for antimicrobial resistance. As a result, this study shows that the *K. rhizophila* strains isolated from *S. munzuricus* possess a species specific and limited resistance profile shaped by environmental selection. The study reveals that advanced genomic and functional research is needed to elucidate the molecular mechanisms of these resistance characteristics and their transfer potential.

## Author Contributions

**Bulent KAR:** conceptualization, investigation, writing – original draft, methodology, validation, visualization, software, formal analysis, data curation. The author has read and approved the final manuscript.

## Funding

The author has nothing to report.

## Ethics Statement

The author has nothing to report.

## Conflicts of Interest

The author declares no conflicts of interest.

## Data Availability

The data that supports the findings of this study are available in the supporting information of this article.
